# End-of-Life Care in Acute Hospitals: Practice Change Reported by Health Professionals Following Online Education

**DOI:** 10.3390/healthcare8030254

**Published:** 2020-08-06

**Authors:** Deb Rawlings, Huahua Yin, Kim Devery, Deidre Morgan, Jennifer Tieman

**Affiliations:** Palliative and Supportive Services, Flinders University, Adelaide, SA 5001, Australia; huahua.yin@flinders.edu.au (H.Y.); kim.devery@flinders.edu.au (K.D.); deidre.morgan@flinders.edu.au (D.M.); jennifer.tieman@flinders.edu.au (J.T.)

**Keywords:** hospital, end of life, e-learning, practice change, healthcare professionals, education

## Abstract

Providing quality care for those dying in hospital is challenging for health professionals who receive little training in this. “End of Life Essentials” (EOLE) was developed to address gaps in health professionals’ knowledge, skills and confidence in end-of-life care via the provision of online learning modules and practice resources. This study aimed to determine whether respondents could describe clinical practice change as a result of module completion. Deidentified data were collected between October and November 2018 from learners registered for the online learning modules. Both quantitative and qualitative data were extracted and analysed. The survey design and conduct were reviewed, and ethical approval was obtained. Although the response rate was very low, results from *n* = 122 learners show improvements in knowledge, skills, awareness and confidence as a result of the undertaking of the learning modules. Two thirds self-reported practice changes (71%, *n* = 59) following the education, with “communication” cited most commonly (*n* = 19). The findings suggest that the EOLE education modules can help to improve end-of-life care by increasing health professionals’ awareness of good practice as well as their knowledge, skills and confidence. Online learning has also been reinforced as an appropriate forum for end-of-life education. Following education, implementing what has been learned occurs more easily at a personal level rather than at a team and organisational level. Barriers to and enablers of clinical practice change in hospital are described, including the fact that the organisation may not be responsive to changes or have the relevant resources to support change.

## 1. Introduction

Increasing numbers of people die in acute care settings [1,2,3], often in Western or developed countries where this can be as high as 50% [2,4]. In addition, most people will visit an acute hospital in their last year of life [5], with hospitals by default becoming the settings where even expected deaths take place [4]. Health professionals in acute hospitals are largely unprepared to manage the care of those who are dying, except for those who have undertaken further/specialist/postgraduate training around end-of-life care [2,3]. Undergraduate degrees do not address end-of-life care in sufficient detail [6], and an inadequately trained workforce poses a major barrier to the provision of quality end-of-life care, with examples found in medicine/nursing [7] and allied health [8]. The consequences of this are that health professionals—and as a result, families—can be unprepared for a death, possibly leading to adverse bereavement outcomes [2].

In 2015, the Australian Commission on Safety and Quality in Health Care (ACSQHC) released the *National Consensus Statement on Essential Elements for safe and high-quality end-of-life care* (The Consensus Statement) [9], containing recommendations for clinical practice in end-of-life care. End of Life Essentials (EOLE) (https://www.endoflifeessentials.com.au/), an Australian Government-funded project, developed online education modules based on the Consensus Statement [9], which addresses the educational needs of health professionals in acute hospitals [10]. Six free online education modules for health professionals working in acute hospitals were launched in the latter part of 2016: Dying, a normal part of life; patient-centred communication and decision-making; Recognising the end-of-life; Planning end-of-life care—Goals of care; Teams and continuity for the patient; and, Responding to concerns. Asynchronous online learning is used to provide education to anyone, anywhere, at any time, and has been found to be no less effective than traditional learning [11,12], although this can be situational [13]. Online learning is widely and increasingly used for health professional education as well as for mandated updates required by organisations to overcome barriers to accessing face to face education, such as backfill, time and cost. Online learning modules are accessed by health professionals for Continuing Professional Development (CPD) purposes [14], for their own interest, as a precursor to more formal palliative care education, or as a preparation for audit and hospital accreditation. Assessment against the Australian National Safety and Quality Health Service (NSQHS) standards commenced in January 2019 [15].

The health professional registration number for the modules at the end of 2018 was 8598, with 11,384 module completions, indicating that they are being used and accessed, although not all modules will be completed by all learners. The evaluation of each module (pre-test and post-test) has been included to formally measure change in end-of-life care knowledge and is not reported here. Transfer of training [16], transfer of learning [17], knowledge translation [18] and knowledge to action [19], are all examples of models or frameworks developed to address and evaluate the transfer or translation of what is learned into practice or to the workplace. It is evident from the work undertaken in this space that there are known barriers to and enablers of implementing what has been learned in the reality of a busy clinical environment [20]. The authors are involved in ongoing research to look at ways of addressing these barriers to practice change in end-of-life care in hospitals.

Changes in practice can be formal (e.g., initiated organisational wide/structured) or informal (e.g., change or changes at the individual level mimicked by others). The former are more easily identifiable as practice change while the latter are often subtle [21]. It can also be difficult to identify what change has occurred as a result of informal education activities such as CPD, as measuring change is not always its intent [22]. The EOLE project has undertaken a range of data collection activities to explore and describe possible changes in knowledge, skills and behaviours. In the EOLE education modules, practice change concepts were included from inception, targeted at the individuals taking them. For example, at the end of each EOLE module, a question was posed: “*Tomorrow, the one thing I can change to more appropriately provide end-of-life care is…*” [23]. An implementation toolkit was also developed to facilitate changes in practice more broadly [10], all intended to allow the tracking of potential practice change.

In order to understand what is occurring in practice as a result of the EOLE education, an online survey specific to the learning modules was developed and administered to those who had registered for the education. The aim was to determine whether respondents reported a change in clinical practice as a result of specific module completion. The survey was not linked to the content of the training (the pre-test post-evaluation does this) but rather looked at whether the education had played a part in clinical practice change. Self-reported change has been measured in two ways, (1) quantitatively via reported influences on clinical practice (changes in awareness, knowledge, skills, confidence and motivation) and (2) qualitatively via self-reported changes in practice.

## 2. Materials and Methods

Ethical approval for the project was provided by Flinders University Social and Behavioural Research Ethics Committee (Project 7012). A modification in relation to this survey was approved on 22 October 2018.

### 2.1. Survey Administration

While the education modules were based on the Consensus Statement [9], the survey itself was based on previous work undertaken on practice change with postgraduate palliative care alumni [24], and on work undertaken on practice change as a result of earlier evaluation of the EOLE education modules [23]. In-house testing of the online survey was completed by the investigators and others in the project group who were unfamiliar with the survey. We acknowledge that this is not representative of the target population. Face validity was assessed by the in-house testing process, and minor changes were made based on the feedback from participants. The survey was administered electronically to those registered with EOLE (*n* = 7455) on 30 October 2018 via the EOLE newsletter mailing list. The electronic newsletter included an invitation via an embedded survey link. A participant information sheet was also provided. The opening rate for this newsletter was 37.6% (*n* = 2802), which indicates that a substantial number of newsletter recipients did not become aware of the survey as they did not read the newsletter. Of those who opened the newsletter, *n* = 153 (5.5%) then clicked the link to the survey.

As the survey was not sent to potential participants directly, unique identifiers could not be used, and an individual could theoretically submit more than one survey response. Respondents completed the online survey anonymously, and no identifying information was collected. The survey included 5 demographics questions, 5 open-ended questions, (qualitatively analysed) 2 yes/no questions with options to provide comments, and 2 statement agreement matrices (quantitative data) Consent was implied upon the completion of the survey. The survey closed on 26 November 2018, with a reminder included in a newsletter on 7 November 2018. The survey design and conduct were reviewed against the Checklist for Reporting Results of Internet E-Surveys (CHERRIES) checklist [25], to address compliance requirements in relation to the survey design, ethics approval, informed consent, survey development and pre-testing, recruitment process, participant sample description and survey administration. Additionally considered were the response rates, prevention of multiple entries from the same individual and data analysis [26].

### 2.2. Quantitative Data Analysis

Changes in clinician awareness, knowledge, skills, confidence and motivation were reported via responses to statements (Strongly Agree to Strongly Disagree). One hundred and twenty-three responses were received. To safeguard integrity, the data from one respondent were excluded via post hoc screening due to the nature of the qualitative comments, which were nonsensical [27]. Data from 122 responses were analysed, but not all surveys were complete. The quantitative data were analysed using SPSS (25.0, IBM, Armonk, NY, USA). Descriptive statistics included medians and interquartile ranges (IQR) for continuous variables because of non-normal distributions, and frequencies and proportions for categorical variables.

### 2.3. Qualitative Data Analysis

Two researchers analysed the qualitative free-text responses provided to questions related to practice change [28]. The questions were *What did you learn from the EOLE modules that has been most important to your care?* and *Can you please tell us more about the practice change?*. The thematic content analysis was deductive [29]. The survey questions informed the first stage of analysis (open coding) where each free-text response was interrogated and deconstructed line by line, and codes were applied. This is considered a “participant”-generated theoretical approach to data [30]. Axial coding (relating codes to each other) was employed to guide the second stage of analysis to develop final codes and subsequent themes [31]. This was applied to the data generated. The two researchers worked to reach consensus in any instances where differences in interpretation were found and in an effort to represent learners’ responses without introducing bias [30].

## 3. Results

The majority of the respondents were female (89.2%, *n* = 66), with a median age at 54.5 years (IQR: 49.0–60.0). By profession, 73% (*n* = 54) of the respondents were nurses, while 20.3% (*n* = 15) and 6.8% (*n* = 5) were allied health professionals and doctors, respectively. The median length of time spent working in their occupational roles was 20.0 years (IQR: 10.0–30.0). Over half of the respondents worked in hospitals (61.3%, *n* = 46).

The majority (68.9%, *n* = 84) of the respondents completed at least one module, with 42.9% (*n* = 36) completing all six modules. Module popularity was not calculated, as learners undertook modules in the order in which they appeared on the website. Of those who did not complete any modules, 84.2% (*n* = 16) reported that they did not have time. (Table 1) Respondents were not prevented from responding to further survey questions if they answered that they could not remember whether they had completed a module or if they answered “no” to the question “Have you completed any EOLE modules?”, as they may have started modules but not completed them.

### 3.1. Influences on Clinical Practice (Awareness, Knowledge, Skills, Confidence and Motivation)

Overall, the majority of the respondents strongly agreed/agreed (60.7%, *n* = 51; 33.3%, *n* = 28) that EOLE modules are important in building awareness of knowledge of end-of-life issues in hospitals. The majority of the respondents reported that the module helped them with their end-of-life care knowledge and skills. Specifically, 45.8% (*n* = 38)/33.7% (*n* = 28) strongly agreed/agreed that the EOLE modules helped them to recognise areas for improvement or change in their own practice, and 42.2% (*n* = 35)/41.0% (*n* = 34) strongly agreed/agreed that the EOLE modules helped them to recognise areas for improvement or change at their workplace. Additionally, 44.6% (*n* = 37)/41.0% (*n* = 34) of the respondents strongly agreed/agreed that the EOLE modules improved their knowledge to make a change, while 45.1% (*n* = 37)/35.4% (*n* = 29) strongly agreed/agreed that the EOLE modules improved their skills in end-of-life care. In addition, 47.6% (*n* = 39)/35.4% (*n* = 29) of the respondents strongly agreed/agreed that the EOLE modules made them more confident in raising end-of-life discussions at work, and 43.9% (*n* = 36)/36.6% (*n* = 30) strongly agreed/agreed that the EOLE modules made them more confident in identifying patient needs. Furthermore, 47.6% (*n* = 39) respondents strongly agreed and 26.8% (*n* = 22) respondents agreed that the EOLE modules helped to motivate them to make a change in practice. However, 18.5% (*n* = 15) and 29.6% (*n* = 24) of the respondents strongly agreed/agreed that they had introduced changes in their workplace because of EOLE, while 42.0% (*n* = 34) held a neutral opinion.

Participants were asked, “*Is it difficult to change end-of-life care?*” (in their own practice, in the team in which they work or in their organisation). Those who answered “yes” were then asked to report against a set of statements. At the personal level, the majority of the respondents strongly disagreed /disagreed that they did not feel that they had the knowledge (21.4%, *n* = 3; 50.0%, *n* = 7)/skills (21.4%, *n* = 3; 42.9%, *n* = 6)/motivation (35.7%, *n* = 5; 42.9%, n = 6) to make any changes in their own practice, and half of the respondents strongly disagreed/disagreed (21.4%, *n* = 3; 28.6%, *n* = 4) that they did not feel that they had confidence in making any changes.

At the team level, similarly, the majority of the respondents strongly disagreed/disagreed that they did not feel that they had the knowledge (17.9%, *n* = 5; 53.6%, *n* = 15)/skills (14.8%, *n* = 4; 51.9%, *n* = 14)/confidence (14.3%, *n* = 4; 42.9%, *n* = 12) to make any changes in the team which they worked. However, the majority of the respondents strongly agreed/agreed that their colleagues may not have supported the changes (14.3%, *n* = 4; 53.6%, *n* = 15), and over half of the respondents indicated that they were not in a role for making changes (10.7%, *n* = 3; 42.9%, *n* = 12).

At the organisation level, over half of the respondents strongly agreed/agreed that the organisation was not responsive to changes (8.2%, *n* = 4; 42.9%, *n* = 21), and half of the respondents strongly agreed/agreed that they were not in a role where they could make changes in the organisation (8.3%, *n* = 4; 41.7%, *n* = 20). Additionally, 10.4% (*n* = 5)/31.3% (*n* = 15) of the respondents strongly disagreed/disagreed that the organisation did not have the relevant resources to make changes, while 2.1% (*n* = 1)/33.3% (*n* = 16) of the respondents strongly agreed/agreed with this statement. In addition, the proportions of the respondents that maintained neutral opinions ranged from 10.7% (*n* = 3) to 32.7% (*n* = 16) in relation to the barriers indicated at personal, team and organisation levels.

### 3.2. Changes in Practice

*(A) “What did you learn from the EOLE modules that has been most important to your care?”*

Responses from 50 respondents were analysed. Codes were developed, and seven themes were subsequently generated (Table 2): (1) Communication (*n* = 24), (2) Improve skills/knowledge (*n* = 9), (3) Express myself/Confidence (*n* = 6), (4) Patient-centred care (*n* = 5), (5) Raising awareness/understanding issues (*n* = 4), (6) Teams (*n* = 4), and (7) Goals of care (*n* = 2).

Nine respondents also made general comments (e.g., *“I loved everything about these modules”).*

Respondents were then asked if they had changed any aspects of their practice following the education. This could be at a personal level, a change within the team, or a change within the organisation. The majority (71.1%, *n* = 59) reported that they had changed their practice with patients and families following the education. While 28.9% (*n* = 24) of the respondents reported that they had not made practice changes, 66.7% (*n* = 16) of these indicated that they intended to make changes to their practice following their learning.

(B) If yes, can you please tell us more about the practice change?

Of *n* = 42 comments, communication was again cited as the most common change in clinical practice (Table 3). The responses were coded as follows: (1) Communication (*n* = 19), (2) Express myself/Confidence (*n* = 5), (3) Patient-centred care (*n* = 5), (4) Teams (*n* = 5), (5) Organisational change (*n* = 3), (6) Teaching others (e.g., colleagues) (*n* = 3), and (7) Improve skills, knowledge (*n* = 2). Dissemination (*n* = 1), patient education (*n* = 1) and self-care awareness (*n* = 1) were also mentioned. One participant made a general comment.

Some respondent comments also demonstrated that practice change could impact patient and family outcomes, highlighting the clinical benefit of changing health professional awareness and practice:

*“All further professional development improves my skills level and enhances my ability to provide my patients with benchmark standard of care”.*

*“Recognising & addressing end-of-life care needs has huge benefits for patients’ peace and comfort (&families &staff) i.e., Outcomes”.*

Participants were then asked, “*Is it difficult to change patient end-of-life care*?”, and 79.7% (*n* = 63) of the respondents answered “no” to changing their own practice while 61.3% (*n* = 46) responded “no” to practice change in the team in which they worked, whereas the majority (65.8%, *n* = 50) of the respondents said “yes” to practice change in their organisation (Table 4).

If respondents indicated “no” to difficulty in changing end-of-life care, they were asked:

(C) Please say what enables practice change to take place.

Forty-four comments were analysed; below are some examples from the responses:

*“Commitment to best practice”*

*“knowledge has a massive impact on changes and it provides a platform to be able to argue what is important to achieve these changes”*

*“It is also beneficial if the team and organisation are supportive to change and willing to listen”*

*“Leadership. Passion. Allies in high places, positive can do attitudes. Less bureaucracy. And not just more documentation!”*

## 4. Discussion

Caring for people with end-of-life care needs in hospitals is challenging for those who lack the knowledge, confidence and skills to support safe and quality care [2]. In this study, we were seeking to examine two self-reported aspects: (1) changes in awareness, knowledge, skills, confidence and motivation, and (2) changes in practice. The responses suggest that the modules helped health professionals to recognise areas for improvement, increased confidence in raising end-of-life discussions with patients and helped to better identify patient needs. Respondents also reported that they felt motivated to make changes in their clinical practice. However, while the responses received provide valuable insight into the role that the EOLE modules play in building awareness and knowledge of the end-of-life issues faced by health professionals in hospitals, a poor response rate limits the generalisability and reliability of these findings.

The population approached to participate was substantial at over 7000 and was extremely targeted as it was people who had registered to undertake EOLE modules. Those who did provide responses are therefore representative of the population of interest. However, the low response rate does raise questions about both the interpretation and future planning for feedback and evaluation. At this level of response, there is the potential for a sampling bias where non-response is unequal among the participants regarding the use of the modules and/or the outcomes associated with the use of the modules. It is not clear from the sample of responders whether they are typical of the cohort or a subset of early adopters. The self-reports may therefore not cover the true range of attitudes, experiences and behavioural outcomes after participation in the modules.

Data collection remains a pragmatic concern for those needing to assess and report on projects and, in this case, contributed to the smaller-than-anticipated response rate. Reviewing the possible causes and impacts of low response rates will be critical in planning for future data collection and evaluation.

Despite these reservations, the respondents were health professionals who chose to undertake further study online. This mode of learning has become an effective way for health professionals to upskill in areas of unmet need [32], and end-of-life care is no exception [33], as discipline-specific undergraduate degrees do not provide comprehensive training about the fundamental basics of palliative care [8] While there is not a strong body of evidence to support online leaning as a vehicle for behaviour change [12], some research [20,34] has shown that small changes at the individual level can and do occur. Students can lack motivation in web-based distance-delivered learning, with higher attrition rates [35], although our results suggest that this is not always the case, with nearly half of the respondents completing all six modules and nearly a quarter completing between three and five. Module completion may be related to their interest in the content, with a lack of time the main stated reason for the non-completion of modules in our study.

The majority of the respondents indicated that they had changed their practice following the education, and sixteen out of the twenty-four respondents who had not changed practice indicated that they intended to change their practice. Intent has been considered a proxy for behaviour change [36], although intent can be affected by external factors such as organisational culture [33], which was evident in our findings.

The majority of the respondents strongly agreed/agreed that EOLE education had motivated them to make a change in practice, consistent with reported increases in knowledge, skills, confidence and the ability to recognise what needed to change. Self-efficacy, or a belief in one’s own abilities, is an important factor in understanding and predicting behavioural change, and can impact people’s thoughts, motivation and performance [37]. Self-efficacy is described [38] as being active between the acquisition of knowledge (such as via module completion or the number of modules completed) and the generation of intent to change practice, mediated by the motivation to change. This, along with perceived barriers to implementation [38], impacts each individual health professional’s practice-change behaviour. Self-efficacy could also determine a person’s views as to whether they have a voice in their team or organisation and whether they are in a role that could influence change. Despite this, not quite half strongly agreed/agreed that they had introduced changes into the workplace following module completion.

The survey results indicate that changing one’s own practice can be achieved more easily than changing team or organisation practice (where change can be hamstrung by others), perhaps due to workplace culture [39]. Organisations not responsive to change limited respondents’ ability to change clinical practice. Five organisational barriers to implementing evidence-based practice (EBP) have been identified [38]: workload, a lack of resources, a lack of authority to change practice, a workplace culture resistant to change, and other staff/management not being supportive of EBP. These were found to be beyond the control of individual clinicians, impacting their ability to change practice despite motivation and competence in EBP.

A major barrier to good end-of-life care in hospital is inadequate knowledge about providing end-of-life care or inadequate access to end-of-life education [40,41]. This came up consistently across our survey as a professional development need with respondents noting education or training as an enabler that could bring about positive change. It must be acknowledged, though, that while EOLE education may help to empower health professionals to change their own practice, they are often powerless to make broader changes in the team or workplace, regardless of the education they have received [25]. This has been described in our study at personal, team and organisational levels. Conversely, identified barriers to change can also be enablers and here include communication, defined as the cornerstone of good practice in end-of-life care [40]. Positive communication [42] and collaboration [43] will enable good care, while a lack of communication will hinder it [39], with often unclear and sometimes contradictory messages conveyed [44].

### 4.1. Limitations

There were limitations to this study. Firstly, the response rate was very low and cautious interpretation is warranted. This might cause non-response bias, with the results not considered to be generalisable [45], although generalisability was not the intent here.

Secondly, the survey was not sent to potential participants directly, due to ethical considerations, but rather by a newsletter (that they had to open to find the survey), which has contributed to the low response rate. The short time frame for survey completion (1 month) is also a limitation, and any future surveys would see this extended to obtain a larger sample size. A lack of time or willingness to complete the online survey might be other contributing factors [46]; however, low response rates are not uncommon when employing online surveys to collect data [47,48,49].

In relation to response bias, comparison with non-survey data retrieved via regular evaluation indicates that the survey responses are valid (similar to the norm) and therefore should not be discounted [46]. It should also be noted that the proportions of the different health professionals (doctors, allied health professionals and nurses) presented by the demographic data of this survey study were consistent with the proportions in the EOLE learner registration data, indicating that this sample may be representative of the EOLE-registered learners. A further limitation is the use of self-reporting by learners. Intent to change, while a proxy for behaviour change, is just that, and the study design did not allow us to ascertain if behaviour change took place.

In addition, this study employed a post-training evaluation design, which may not be able to quantify the changes compared to the pre-post-training evaluation design. However, this exploratory survey focuses on understanding the influence of EOLE on learners’ end-of-life care practice changes based on a combination of quantitative and qualitative data. The questions in the survey provided a context as “the EOLE modules…”; therefore, the results could reflect the self-reported influence of EOLE module learning.

### 4.2. Future Directions

This was a snapshot of learners at one time point and has provided the basis for more robust ongoing evaluation, Future investigations could also include responses to modules by individual health professions (e.g., nurses, doctors and allied health professionals) and investigations into organisational barriers to end-of-life care. Nationwide qualitative research in relation to EOLE learners’ practice changes will be conducted by the research team. The role of champions [50] in advocating for and promoting end-of-life care is an important consideration and one that could also be a way of addressing organisational factors that impinge on learning.

## 5. Conclusions

Increasing numbers of deaths occur in hospital settings, and there is an imperative to ensure that health professionals have adequate training to provide the required care. This exploratory cross-sectional survey study helped to better elucidate learners’ perceptions about the influence of EOLE-module learning and practice changes. Our results show that education heightens individual health care professionals’ awareness, knowledge, confidence and skills in end-of-life care. The majority of the health care professionals reported that they had changed their practice following the online education. In addition, most health professionals did not report that the change to their own practice was difficult. However, they noted that it was more difficult to change end-of-life care within their team or organisation, recognising that organisational and team structures influenced their ability to practice and to create practice change. This study can be seen as a snapshot of the context in which some health professionals are changing their behaviour and clinical practice as it relates to end-of-life care and is part of a larger body of work.

## Figures and Tables

**Table 1:** End of Life Essentials (EOLE) module completion.

	*n*	%
Have you completed any EOLE modules? (*n* = 122)		
No	19	15.6
Yes	84	68.9
I’m not sure	19	15.6
Reasons for not having completed an EOLE module (*n* = 19)		
I have not had time	16	84.2
I forgot	1	5.3
Other	2	10.5
How many modules have you completed? (*n* = 84)		
1	8	9.5
2	9	10.7
3–5	19	22.6
6	36	42.9
Not sure/Don’t know/Can’t remember	12	14.3

**Table 2:** Exemplars of learnings important to care categorised by theme.

Themes	*n*	Examples
Communication	24	*Communication strategies, ways of exploring questions and being ok with not knowing the answers!*
Improve skills/knowledge	9	*Recognizing signs*
Express myself/Confidence	6	*Confidence to have the conversation*
Patient-centred care	5	*I have gained much knowledge in changing end of life from a clinical model to a person centred model.*
Raising awareness/understanding issues	4	*Raising awareness and understanding*
Teams	4	*Also the importance of the team and of setting*
Goals of care	2	*Clear goals of care all together*

**Table 3:** Exemplars of learnings important to care categorized by theme.

Themes	*n*	Examples
Communication	19	*“Being more proactive listening to the patient”*
Express myself/Confidence	5	*“I am more confident to raise end of life issues with clients”*
Patient-centred care	5	*“Making sure that the focus remains on the patient while also supporting the family”*
Teams	5	*“Encouraging discussion at a team level”*
Organisational change	3	*“Completion of the EOLE modules has now been incorporated into the Professional Development Pathways for nurses in our organisation.”*
Teaching others (e.g., colleagues)	3	*“Teaching Students and Interns about EOL Essentials”*
Improve skills/knowledge	2	*“Identifying needs earlier, and being more confident & able to plan quality care”*
Dissemination	1	*“More knowledge sharing”*
Patient education	1	*“I have introduced end of life issue and advanced care planning eductaion (sic) sessions for our patients in pulmonary rehabilitation”*
Self-care awareness	1	*“Self care awareness”*

**Table 4:** Self-reported barriers to practice changes at personal, team and organisation levels.

Questions and Statements	Strongly Agree *n* (%)	Agree *n* (%)	Neutral *n* (%)	Disagree *n* (%)	Strongly Disagree *n* (%)	Total *n*
**Personal Level**						
Is it difficult to change end-of-life care? In your own practice (*n* = 79) *n* (%) No 63 (79.7) Yes 16 (20.3)						
I do not feel that I have the knowledge to make any changes	1 (7.1)	0 (0.0)	3 (21.4)	7 (50.0)	3 (21.4)	14
I do not feel that I have the skills to make any changes	1 (7.1)	1 (7.1)	3 (21.4)	6 (42.9)	3 (21.4)	14
I do not feel that I have the confidence to make any changes	1 (7.1)	4 (28.6)	2 (14.3)	4 (28.6)	3 (21.4)	14
I do not feel that I have motivation to make any changes	0 (0.0)	1 (7.1)	2 (14.3)	6 (42.9)	5 (35.7)	14
**Team Level**						
Is it difficult to change end-of-life care? In the team in which you work (*n* = 75) *n* (%) No 46 (61.3) Yes 29 (38.7)						
I am not in a role where I can make changes in the ward/unit/team	3 (10.7)	12 (42.9)	3 (10.7)	8 (28.6)	2 (7.1)	28
I do not feel that I have the knowledge to make any changes	1 (3.6)	1 (3.6)	6 (21.4)	15 (53.6)	5 (17.9)	28
I do not feel that I have the skills to make any changes	1 (3.7)	3 (11.1)	5 (18.5)	14 (51.9)	4 (14.8)	27
I do not feel that I have the confidence to make any changes	1 (3.6)	3 (10.7)	8 (28.6)	12 (42.9)	4 (14.3)	28
My colleagues may not support the changes	4 (14.3)	15 (53.6)	5 (17.9)	3 (10.7)	1 (3.6)	28
**Organisation Level**						
Is it difficult to change end-of-life care? In your organisation (*n* = 76) *n* (%) No 26 (34.2) Yes 50 (65.8)						
The organisation is not responsive to changes	4 (8.2)	21 (42.9)	16 (32.7)	7 (14.3)	1 (2.0)	49
I am not in a role where I can make changes in the organisation	4 (8.3)	20 (41.7)	9 (18.8)	12 (25.0)	3 (6.3)	48
The organisation does not have the relevant resources to make changes	1 (2.1)	16 (33.3)	11 (22.9)	15 (31.3)	5 (10.4)	48
